# Supplementary material for: Predicting visual function by interpreting a neuronal wiring diagram
Source: Nature. 2024 Oct 2;634(8032):113–23. doi: 10.1038/s41586-024-07953-5 (PMC11446822; doi:10.1038/s41586-024-07953-5)
Supplement: Supplementary file 6 — CRF and ERF predictions for individual TmY4 and TmY9 cells. Analogous to Supplementary Data 3, but for TmY target types. Shown are the top four monosynaptic pathways, the strongest pathway passing through each of the top ten intermediary types (ranking from Extended Data Fig. 7), and the trisynaptic pathway Tm1–TmY–Dm3–TmY (see the section entitled Prediction of spatial normalization). [file 41586_2024_7953_MOESM6_ESM.zip › DataS4/TmY9qΓèÑ/720575940640388992.pdf]

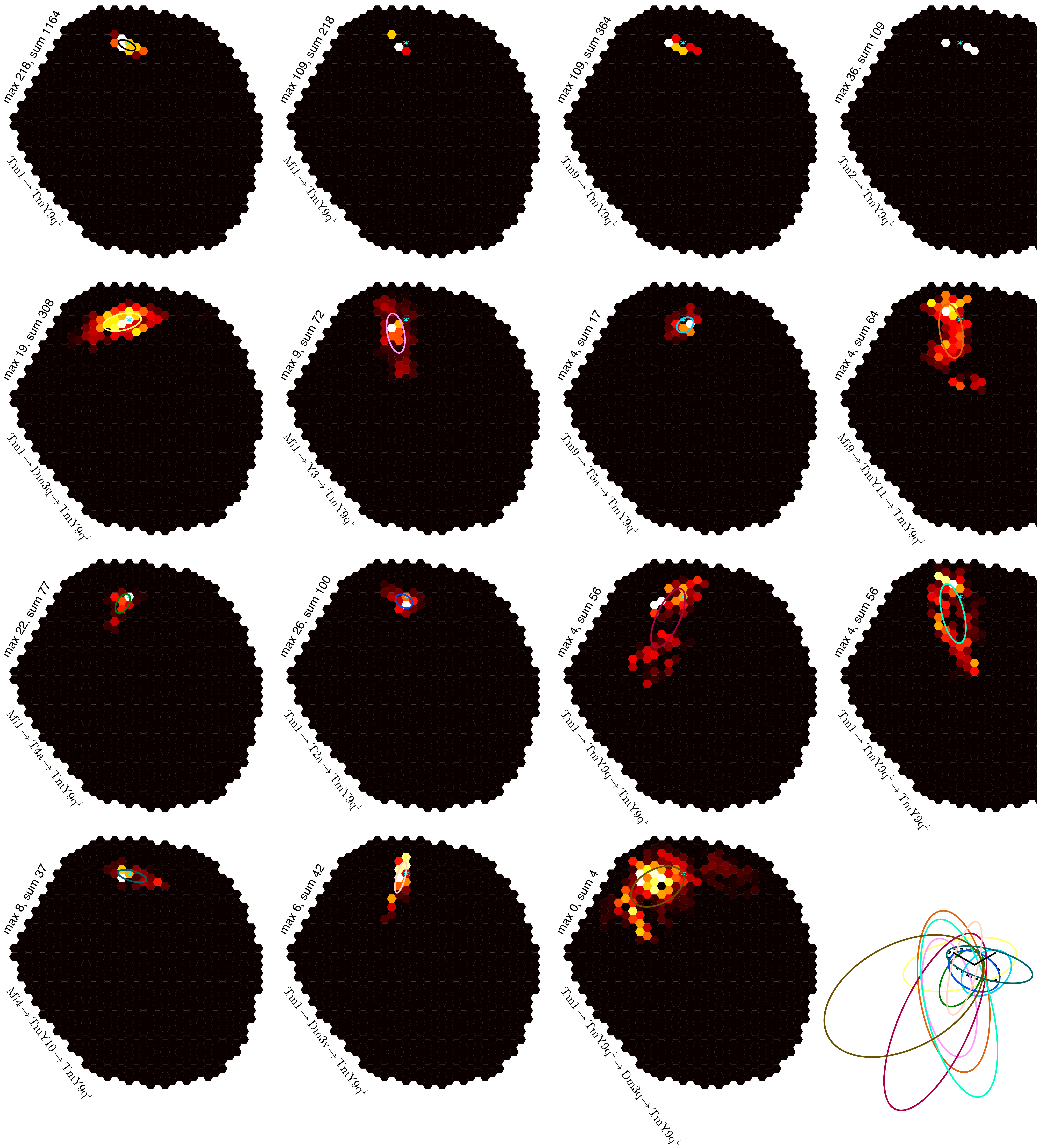
max 218, sum 1164
Tm1 → TmY9q⊥
max 109, sum 218
Mi1 → TmY9q⊥
max 109, sum 364
Tm9 → TmY9q⊥
max 36, sum 109
Tm2 → TmY9q⊥
max 19, sum 308
Tm1 → Dm3q → TmY9q⊥
max 9, sum 72
Mi1 → Y3 → TmY9q⊥
max 4, sum 17
Tm9 → T5a → TmY9q⊥
max 4, sum 64
Mi9 → TmY11 → TmY9q⊥
max 22, sum 77
Mi1 → T4a → TmY9q⊥
max 26, sum 100
Tm1 → T2a → TmY9q⊥
max 4, sum 56
Tm1 → TmY9q → TmY9q⊥
max 4, sum 56
Tm1 → TmY9q⊥ → TmY9q⊥
max 8, sum 37
Mi4 → TmY10 → TmY9q⊥
max 6, sum 42
Tm1 → Dm3v → TmY9q⊥
max 0, sum 4
Tm1 → TmY9q⊥ → Dm3q → TmY9q⊥
